# Autistic Characteristics in a Nationally Representative Clinical Sample of Adolescents Seeking Medical Gender-Affirming Treatment in Norway

**DOI:** 10.1007/s10803-023-06181-6

**Published:** 2023-12-06

**Authors:** Linda W. David, Nina Stenberg, Trond H. Diseth, Sissel Berge Helverschou, Cecilie Bjertness Nyquist, Roald A. Øien, Anne Waehre

**Affiliations:** 1 Department of Child and Adolescent Mental Health in Hospitals, Oslo University Hospital, Oslo, Norway; 2 Division of Paediatric and Adolescent Medicine, Institute of Clinical Medicine, Faculty of Medicine, University of Oslo, Oslo, Norway; 3 Regional Resource Center for Autism, ADHD and Tourette Syndrome, South-Eastern Norway Regional Health Authority, Oslo University Hospital, Oslo, Norway; 4 NevSom - Norwegian Centre of Expertice for Neurodevelopmental disorders and Hypersomnias, Oslo University Hospital, Oslo, Norway; 5 The Arctic University of Norway, UNN - University Hospital of Northern Norway, Tromsø, Norway; 6 School of Medicine, Child Study Center, Yale University, New Haven, USA

**Keywords:** Gender incongruence, Gender dysphoria, Autism spectrum disorder, Social responsiveness scale

## Abstract

**Purpose:**

Several studies have reported on the intersection of autism and gender incongruence (GI) in clinical populations. This study aims to investigate autistic characteristics and registered autism spectrum diagnoses (ASD) in a clinical cohort of 83 adolescents referred to the National Gender Team for Children and Adolescents in Norway during 2020.

**Methods:**

Parents completed the Social Responsiveness Scale (SRS). Background information and registered psychiatric diagnoses were extracted from patient files.

**Results:**

The results showed that 25% of the participants scored within the clinical range on the SRS: 27.4% of adolescents who were assigned female at birth (AFAB) and 19.0% of adolescents who were assigned male at birth (AMAB). AFAB had significantly higher scores on SRS Total Scale and the Social Motivation and Autistic Mannerisms subscales compared to the female norm group. AMAB had higher scores on the Social Motivation subscale and lower scores on the Social Awareness subscale, compared to the male norm population. Information from patient files revealed that 67.5% had one or more registered psychiatric diagnosis. 9.6% had received an ASD diagnosis, all AFAB. 18.1% had received an attention deficit hyperactivity disorder (ADHD) diagnosis. The most common psychiatric diagnoses were depression (25.3%) and anxiety disorders (18.1%). Further, 44.6% had a history of self-harm, and 15.7% had a history of a suicide attempt.

**Conclusion:**

The results showed an overrepresentation of ASD diagnoses and autistic characteristics measured by SRS for AFAB. There was an overrepresentation of psychiatric diagnoses for both the AFAB and the AMAB group in this study sample. Implications for treatment and future research are discussed.

The prevalence of autism spectrum disorders (ASD) is increasing both in Norway (Isaksen et al., 2012; Surén et al., 2019) and internationally (Atladottir et al., 2015; Bölte et al., 2023; Idring et al., 2015; Maenner et al., 2023). Studies suggest that females receive ASD diagnoses later than males (Loomes et al., 2017; Posserud et al., 2021). There are also increasing numbers of adolescents seeking treatment at gender identity services in Western countries (Bouman et al., 2016; Kaltiala et al., 2020; Zucker, 2017). Over the last decade, there has been a shift in the sex ratio from an overrepresentation of persons assigned male sex at birth (AMAB) to an overrepresentation of persons assigned female sex at birth (AFAB), often referred after the beginning of puberty (Arnoldussen et al., 2022; de Graaf et al., 2018; Expósito-Campos et al., 2023). The reasons for the increase in referral rates to gender clinics and the shift in sex ratio remain unknown (Bewley et al., 2019; Dhejne, 2017; Kreukels & Guillamon, 2016).

Gender incongruence (GI) in adolescence is defined as the experience of a persistent and marked mismatch between one’s assigned sex at birth and their gender identity (World Health Organization, 2018). This experience often leads to a strong inclination to live as the gender they identify with, by socially transitioning and for some by receiving gender-affirming interventions, such as hormonal treatment, surgery, or other healthcare services. In the Diagnostic and Statistical Manual of Mental Disorder (American Psychiatric Association, 2022), the psychological distress or emotional pain associated with this mismatch is usually labeled gender dysphoria (GD). Other terms used are opposite sex identification and transgender identity, consisting of various definitions of gender diversity including non-binary identification. In this paper, the terms AFAB (persons assigned female at birth) and AMAB (persons assigned male at birth) will be used.

In previous years, a growing number of studies have highlighted the intersection of ASD and GI (Heylens et al., 2018; Kallitsounaki & Williams, 2022; Skagerberg et al., 2015; van der Miesen et al., 2018b; Øien et al., 2018). It has been suggested that adolescents who experience the co-occurrence of ASD and GI might benefit from gender-affirming treatment (GAT) (Strang et al., 2018). However, the overlapping symptoms in these conditions make assessing initiation of GAT more complex, and it can become challenging to determine whether GAT is the best intervention for the individual at the current time (van der Miesen et al., 2018a; Miesen et al., 2018b).

Autistic people typically have difficulties with communication and social interaction and may display patterns of focused interests and behavior (APA, 2022). As such, autistic people may struggle to communicate their thoughts and emotions and tend to have a concrete or “black-and-white” style of thinking. Furthermore, they typically experience difficulties with executive functions, such as flexibility, planning, and future thinking (Demetriou et al., 2018). They frequently meet barriers in society such as a lack of flexibility in the educational system and work environments. The intersection of ASD and GI requires clinical awareness (Bouzy et al., 2023). Clinical guidelines for co-occurring ASD and GD suggest an extended diagnostic period and the use of visual materials such as checklists and flowcharts to ease communication and understanding (Strang et al., 2018). It is, however, important to individualize assessment as autistic persons are all different. Further, it is essential to identify autistic characteristics as early as possible so that necessary adjustments to assessments can be made to improve clinician-client communication. It is also important to acquire a better understanding of the mental health needs in their everyday life so that relevant support can be given to improve their quality of life. The Social Responsiveness Scale (SRS) is a questionnaire that provides quantitative measures to detect autistic characteristics in children and adolescents (Constantino, 2005). Also mild autistic characteristics could influence social functioning.

Studies in other Western countries have found an overrepresentation of ASD diagnoses and features in GI populations (Kallitsounaki & Williams, 2022). So far, no studies on the intersection of ASD and GI have been published in Norway. In this study, we investigated the co-occurrence of ASD and GI in a nationally representative clinical sample of adolescents with GI. Since the growth of referrals to gender clinics in recent years mainly have been driven by an increase in adolescent AFAB (Arnoldussen et al., 2022), we also investigated gender differences. Our aims were as follows: (1) to report on the prevalence of ASD diagnoses and other verified psychiatric diagnoses in a Norwegian sample of adolescents referred for gender-affirming treatment, (2) to identify autistic characteristics as measured by the Social Responsiveness Scale (SRS), (3) to investigate differences in SRS scores in participants who were assigned female at birth (AFAB) versus participants who were assigned male at birth (AMAB) and compare both genders to a general population sample (Constantino, 2005).

## Methods

### Participants and Settings

In Norway, a national gender team (NGT) receive referrals of all patients under the age of 18 years from the entire country, who apply for gender affirming treatment. The NGT is the only public non-profit gender clinic for all inhabitants in Norway providing full gender affirming treatment. All referrals come from local child and adolescent psychiatric outpatient clinics (CAPOC) to the NGT in Oslo. All patients aged 13 to 18 years at the time of referral, with an initial appointment at the NGT in Norway between January and December 2020, were invited to participate in the study. 83 (68.6%) patients agreed to participate. All of them applied for gender affirming treatment, hence the referral to the NGT. All of them were registered with a reported binary gender identity at initial appointment. Of the 83 participants, 62 (74.7%) were AFAB and 21 (25.3%) were AMAB. Mean age was 15.6 years. Exclusion criteria were having an IQ below 70, psychotic illnesses with active symptoms, and adolescents with a minority language background/limited Norwegian language skills. None of the participants had received any medical treatment, such as puberty blockers or gender-affirming hormone treatment, at the time of their inclusion. Inclusion time was during the first year of the COVID 19 pandemic. However, appointments at the NGT were continued as usual. The study is part of a larger project including planned in-depth interviews, where a user representative group is involved.

### Measures

#### Registered Psychiatric Diagnoses, Self-Harm and Suicide Attempts

The following data was extracted from referral letters from the CAPOC: prior and current psychiatric diagnoses, earlier and ongoing self - harm, and reported suicide attempts during lifetime. The NGT is the only publicly funded service offering GAT for persons in Norway, and is the only publicly funded service offering surgery for this group. Before a potential referral to the NGT, all children and adolescents undergo an initial standardized psychiatric assessment at their CAPOC. The assessment at the CAPOC prior to any referral to the NGT, consists of a developmental history interview and a description of the family situation, the schedule for affective disorders and schizophrenia (Kaufman et al., 1997), the Achenbach System of Empirically Based Assessment (ASEBA) (Achenbach, 2014) and a cognitive assessment when indicated by the referring practitioner (Wechsler, 2014). Systematic screening for post-traumatic stress symptoms is done using the Child and Adolescent Trauma Screen (CATS) (Sachser et al., 2017). Following the assessment, the referring practitioner and the team decide if an ICD-10 diagnosis is present or not, before referring to the NGT. Some patients undergoes specific assessments, for example for autism or ADHD, if indicated. The CAPOCs are geographically spread across the country and have to follow the same standardized diagnostic procedures as decided by the health authorities. The 83 participants of this study came from the whole country. They were equally distributed from all four health regions in the country (referrals per 100 000 aged 13–17 years); North n = 11 (39.1); West n = 23 (33.6); South-East n = 62 (35.3) and Mid-Country n = 24 (37.2).

In the referral letters, there were information on the standardized assessment. In addition, the NGT has its own referral routines, which ensures that the same diagnostic tools are used in local assessments. Only diagnosed psychiatric conditions were included in the study, as well as reported self-harm and suicide attempts. The autism assessments included ADOS-2 (Lord et al., 2012) and ADI-R (Rutter et al., 2003) assessment tools. IQ measures were also included in these assessments, with WISC-V (Wechsler, 2014) or WAIS-IV (Wechsler, 2008).

Birth-assigned gender and age at the time of referral were also recorded.

### Social Responsiveness Scale (SRS)

The SRS is a questionnaire completed by parents or teachers that measures social challenges and autistic features in children and adolescents aged 4 to 18 years (Constantino, 2005). This study used a parental report, and the questionnaire was completed by one of the parents. The questionnaire has 65 statements (e.g., ‘seems more fidgety in social situations than when alone,’ and ‘would rather be alone than with others’) scored on a 4-point scale ranging from 1 = not true to 4 = almost always true. It includes five subscales: Social Awareness, Social Cognition, Social Communication, Social Motivation, and Autistic Mannerisms. The total raw scores can be transformed into t-scores to provide normative positions. The overall score indicates whether the person falls within the normal range (t-score below 60), mild/moderate range (t-score of 60—75; typical for young people with ASD and an IQ within the normal range), or severe range of autistic features (t-score 75 or higher; strongly associated with ASD, Asperger’s Syndrome, and severe PDD-NOS) (Constantino, 2005). Completion of the parental report takes approximately 15—20 min. The SRS has been validated in the American norm population and is commonly used in clinical settings. Standardization of the SRS is based on a diverse cross section sample of over 1600 children from the general population in the US from a Midwestern state including a large metropolitan area, covering ages from 4 to 18 years. Separate norms are provided for teacher and parent report, respectively, and for gender (males and females). Five different studies contributed cases to the normative sample. Age differences appeared minimal. There were strong differences related to gender, rawscores for males were always higher than for females (Constantino, 2005).

### Statistics

In line with aim one, frequencies and proportions of verified psychiatric diagnoses were analyzed with Chi-square tests (*X*^2^).

In line with the second and third aim, independent sample *t*-tests were conducted to investigate possible gender differences in SRS scores between AFAB and AMAB in the clinical sample. In line with aim three, we calculated t-tests and effect size (Cohen’s d) to compare the SRS scores of AMAB and AFAB in the current sample with SRS scores of males and females from the standardization data of the SRS. Data in line with aim two was analyzed using SPSS Statistics 28. T-tests and Cohen’s d analyses in line with aim three were analyzed using STATA version 17.

## Results

### Registered Psychiatric Diagnoses, Self-Harm and Suicide Attempts

An overview of psychiatric diagnoses, including differences between assigned genders is presented in Table 1. In line with aim one, clinical file data revealed that as many as two-thirds of the participants had received one or more psychiatric diagnoses from their local psychiatric clinic before or at the time of their referral to the NGT. 9.6% had received an ASD diagnosis, and 18.1% received an ADHD diagnosis. All participants with ASD diagnoses were AFAB. The most common psychiatric diagnoses were depression (25.3%) and anxiety disorders (18.1%). 44.6% of the sample had reported prior or ongoing self-harm, and 15.7% had a history of suicide attempts. The prevalence of anxiety disorders and history of suicide attempts was higher in AFAB than AMAB. No significant differences between genders were found, but there were lower numbers of psychiatric diagnoses in the AMAB group.

**Table 1:** Registered psychiatric diagnoses, self-harm, and suicide attempts

Description	Total	Assigned female gender at birth (AFAB)	Assigned male gender at birth (AMAB)	AFAB vs. AMAB p-value
	N = 83	N = 62	N = 21	
One or more psychiatric diagnoses	56 (67.5%)	44 (71.0%)	12 (57.1%)	0.242
F20-F29 Psychotic disorders	2 (2.4%)	2 (3.2%)	0 (0.0%)	1.000
F32 – F33 Depressive episode and recurrent depressive disorder	21 (25.3%)	16 (25.8%)	5 (23.8%)	0.856
F34-F39 Other affective disorders	1 (1.2%)	0 (0.0%)	1 (4.8%)	0.253
F40 – F41 Anxiety disorders	15 (18.1%)	14 (22.6%)	1 (4.8%)	0.067
F43 Reaction to severe stress, and adjustment disorder	10 (12.0%)	9 (14.5%)	1 (4.8%)	0.235
F44-F48 Dissociative disorders, somatoform disorders and other neurotic disorders	1 (1.2%)	1 (1.6%)	0 (0.0%)	1.000
F50 Eating disorders	5 (6.0%)	4 (6.5%)	1 (4.8%)	0.779
F80-F83 Specific and mixed developmental disorders	9 (10.8%)	6 (9.7%)	3 (14.3%)	0.557
F84 Autism disorders	8 (9.6%)	8 (12.9%)	0 (0.0%)	0.083
F90 Hyperkinetic disorders	15 (18.1%)	11 (17.7%)	4 (19.0%)	0.893
F91 Conduct disorders	1 (1.2%)	0 (0.0%)	1 (4.8%)	0.253
F93 Emotional disorders with onset specific to childhood	2 (2.4%)	2 (3.2%)	0 (0.0%)	1.000
F94 Disorders of social functioning with onset specific to childhood and adolescence	1 (1.2%)	0 (0.0%)	1 (4.8%)	0.253
F95 Tic disorders	2 (2.4%)	1 (1.6%)	1 (4.8%)	0.444
History of suicide attempt	13 (15.7%)	12 (19.4%)	1 (4.8%)	0.112
History of self harm	37 (44.6%)	29 (46.8%)	8 (38.1%)	0.489

### SRS Clinical Categories

The T-scores and severity range on the SRS by assigned gender are shown in Table 2. 27.4% of the AFAB and 19.0% of the AMAB scored within the clinical range.

**Table 2:** SRS clinical category by assigned gender

	Normal range n (%)	Mild-moderate clinical range n (%)	Severe clinical range n (%)
Assigned male at birth (AMAB)	17 (81.0%)	4 (19.0%)	0 (0.0%)
Assigned female at birth (AFAB)	45 (72.6%)	6 (9.7%)	11 (17.7%)

*Note*. Normal range = T-score below 60. Mild to moderate range = T-score 60–75. Severe range = T-score 75 or higher

### SRS Scores and Assigned Gender

In line with aim two, independent sample *t*-tests were conducted to investigate possible differences between AFAB and AMAB concerning SRS scores. The results showed no significant difference in total or subscale raw scores (all *p* > 0.05).

### SRS Scores Compared to Norm Populations

**Fig. 1:**
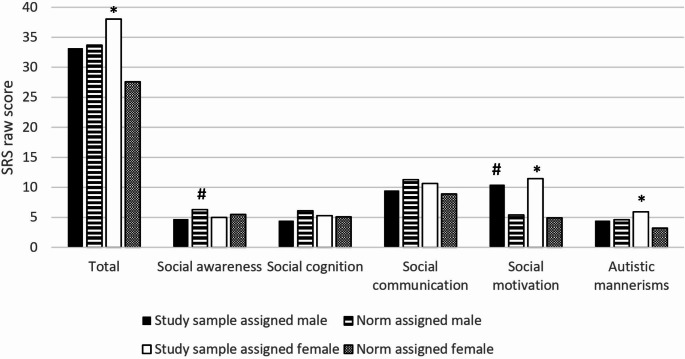
SRS raw score by gender. *Note*. SRS = Social Responsiveness Scale. Norm population provided by Constantino and Gruber, 2005. * = *p < 0.05* study sample assigned female vs. norm assigned females. # = *p < 0.05* study sample assigned male vs. norm assigned males

In line with aim three, independent t-tests and effect sizes were calculated to investigate differences in SRS scores between the clinical sample of AFAB and the female norm group, and between the clinical sample of AMAB and the male norm group, respectively (Constantino, 2005) (Fig. 1, Table 3a and 3b).

**Table 3a:** SRS total and subscale scores by study sample groups and norm groups, assigned male note

SRS score	Study sample assigned male (n = 21) Mean (SD) [Min, max]	Norm assigned male (n = 512) Mean (SD)	Mean difference [95% CI]	*t* (531)	*p*	Cohen’s *d*
Total SRS	33.19 (20.3) [3, 80]	33.7 (20.9)	0.51 [-9.6-8.62]	0.11	0.91	0.02
Social awareness	4.71 (3.5) [1,14]	6.3 (3.1)	-1.59 [-2.95-0.23]	2.29	0.02 *	0.51
Social cognition	4.43 (4.48) [0,16]	6.1 (4.5)	-1.67 [-3.64-0.30]	1.67	0.1	0.37
Social communication	9.48 (7.3) [0,23]	11.3 (7.9)	-1.82 [-5.27-1.62]	1.04	0.30	0.23
Social motivation	10.43 (5.2) [0,19]	5.4 (4.2)	5.03 [3.17–6.89]	5.33	0.001 *	1.19
Autistic mannerisms	4.43 (3.7) [0,12]	4.6 (4.4)	-0.17 [-2.08-1.74]	0.17	0.86	0.04

* *p* < 0.05

**Table 3b:** SRS total and subscale scores by study sample groups and norm groups, assigned female

SRS score	Study sample assigned female (n = 62) Mean (SD) [Min,max]	Norm assigned female (n = 569) Mean (SD)	Mean difference [95% CI]	*t* (629)	*p*	Cohen’s *d*
Total SRS	38.03 (30.7) [2,133]	27.6 (18.1)	10.43 [5.26–15.60]	3.96	0.001 *	0.53
Social awareness	5.00 (4.1) [0,17]	5.5 (2.8)	-0.50 [-1.28-0.28]	1.27	0.21	0.17
Social cognition	5.27 (6.1) [0,25]	5.1 (4.1)	0.17 [-1.00-1.31]	0.29	0.77	0.39
Social communication	10.65 (9.9) [0,38]	8.9 (6.8)	1,80 [-0.13-3.63]	1.83	0.07	0.24
Social motivation	11.44 (6.1) [2,26]	4.9 (4.1)	6,54 [5.40–7.68]	11.28	0.001 *	1.51
Autistic mannerisms	5.94 (6.9) [0,27]	3.2 (3.4)	2.74 [1.72–3.76]	5.28	0.001 *	0.71

*Note*. * *p* < 0.05

A small effect size was found on the total raw scores in the AMAB versus males in the norm population. However, a significant effect was found on the Social Awareness subscale (*p* < 0.02), with a moderate effect size (*d =* 0.51) where the AMAB group had *lower* scores compared to the norm population. In other words, AMAB youth were reported to have greater social awareness than their peers. Furthermore, on the Social Motivation subscale, the AMAB score was significantly higher than the norm population (*p* < 0.001), with a large effect size (*d* = 1.19), suggesting more difficulties with social motivation than their peers.

In the AFAB group, the total SRS scores were significantly higher than the norm population (*p* < 0.001) and had a moderate effect size (*d* = 0.53). In this group, significant results were found on both the Social Motivation subscale (*p* < 0.001) with a large effect size (*d* = 1.51) and the Autistic Mannerism subscale (*p* < 0.001) with a moderate effect size (*d* = 0.71).

## Discussion

The current study aimed to investigate autism diagnoses and autistic characteristics in a nationally representative sample of adolescents with GI on the Norwegian NGT during one year. AFAB scored significantly higher compared to the female norm group on the Social Motivation and Autistic Mannerisms SRS subscales, as well as on the total score. In contrast, AMAB scored significantly higher compared to the male norm group on the Social Motivation subscale, but significantly lower on the Social Awareness subscale. Concerning registered psychiatric diagnoses, a high percentage of the study sample was diagnosed with ADHD, with more cases being AFAB participants. According to assessments at the CAPOC, approximately 10% of the sample had a registered ASD diagnosis (all AFAB), and one in four scored within the clinical range on the SRS at the NGT. This is considerably higher than the prevalence of ASD in the general population, which is estimated to be around 1–2%, (Bölte et al., 2023; Maenner et al., 2023; Lai et al., 2014). However, similar percentages of ASD diagnoses have been reported in other GI study samples (de Vries et al., 2010; Kallitsounaki & Williams, 2022). Several recent studies have revealed the co-occurrence of GI and ASD (Øien et al., 2018). The prevalence of ASD in the GI population differs widely according to the measures used. A recent review states that ASD diagnoses occur in 3 to 21.3% of children and adolescents with GI (Kallitsounaki & Williams, 2022).

Some previous studies of GI samples have found a higher percentage of scores in the clinical range on ASD screening instruments in children, adolescents, and adults compared to the current study (Skagerberg et al., 2015). SRS is not a diagnostic tool for ASD. However, it is commonly used as a screening tool for identifying autistic features. Scores within the severe clinical range are highly associated with ASD diagnoses (Constantino, 2005). Significantly, according to Constantino (2005), mild to moderate clinical range scores are typical for adolescents with ASD who are within the average IQ range.

We found that, compared to AMAB, more AFAB had scores within the severe clinical range, according to the SRS. We also found a significantly higher total SRS raw score for AFAB compared to gender-and age-matched norm data (Constantino, 2005). Generally, it has been argued that ASD in girls is more challenging to detect because they more easily develop social camouflaging strategies that compensate for and mask autistic characteristics during social interactions (Hull et al., 2019). Interestingly, in AFAB, we found higher scores on Social Motivation and the Autistic Mannerism SRS subscales. The high scores on the Autistic Mannerism subscale may indicate more repetitive or stereotyped behaviors in the AFAB GI group compared to a normative general population sample. The higher scores on the SRS Social Motivation subscale for both birth-assigned genders could also involve social withdrawal and a lack of engagement in social activity due to bullying, minority stress, or other difficulties associated with GI. In this study, we found that 18.1% had a diagnosed anxiety disorder. As such, the high scores on this SRS subscale could also be due to social anxiety or gender-related stress (Cholemkery et al., 2014). Some studies have shown that autistic characteristics persist (Nobili et al., 2020), while others have shown a decrease in symptoms after initiating GAT (Mazzoli et al., 2022). A longitudinal study of children with GI found that SRS scores remained stable over time after puberty-suppressing treatment was initiated (Russell et al., 2021). This may indicate that higher SRS scores are related to autistic characteristics rather than GI-related stress.

Interestingly, we found that the AMAB clinical group had lower SRS scores on the Social Awareness subscale than the norm population, meaning greater social awareness than the norm population. They also had significantly higher scores on the Social Motivation subscale. This finding could suggest that the AMAB group was more aware of their social surroundings, and they seemed to have a different SRS profile than AFAB and the norm male group.

The increase in referrals to gender clinics over the last few years has mainly been driven by adolescent AFAB (Arnoldussen et al., 2022; Kaltiala-Heino et al., 2015; Kaltiala et al., 2020). Findings from this study suggest a tendency toward more AFAB showing autistic characteristics compared to AMAB among adolescents seeking medical treatment for GI. Additionally, only AFAB had a registered ASD diagnosis in this sample. Although we did not find significant differences between AFAB and AMAB in past diagnoses, a trend-level finding that is worth drawing attention to is the higher occurrence of ASD, previous suicide attempts and the prevalence of anxiety disorder in AFAB. This is particularly striking for ASD given that the expected finding based on normative samples and assigned sex at birth would be the reverse of an AMAB preponderance of ASD (Bölte et al., 2023; Loomes et al., 2017). Research suggests that level of fetal testosterone exposure is essential for gender role behavior and gender identity (Hines, 2020). Research also suggests that fetal testosterone exposure may be involved in causing autistic traits (Auyeung et al., 2009; Baron-Cohen et al., 2015). Thus, the overlap of GI and ASD in AFAB may be explained by a common biological etiology.

Diagnostic norms for ASD and cut-offs on ASD questionnaires were developed in predominantly male samples. Results from extensive population-based studies show that the average female score on ASD questionnaires is further away from the ASD cut-off than the average male score is, and that females with ASD’ score further away from the female population average than males with ASD score from the male population average (Baron-Cohen et al., 2001; Lundström et al., 2019).

It has been suggested that the development of an identity and sense of self may be different in autistic people due to social and cognitive challenges, such as emotional and social self-awareness (Huang et al., 2017). Socialization processes are influential in developing gender identity—this includes both external socialization and self-socialization (Hines, 2020). Previous studies have shown that “tomboyism” is over-represented in autistic females (Bejerot & Eriksson, 2014; Brunissen et al., 2021). Thus, autistic females might differ from their non-autistic peers in the way they perceive and adapt to female societal gender norms, and their behaviors and interests might be more similar to the male gender norm.

Systematic reviews have suggested that ADHD is more frequent in gender-incongruent individuals than in gender-congruent individuals (Thrower et al., 2020; Warrier et al., 2020). In this study, we found that 18% of the adolescents were previously diagnosed with ADHD, a much higher co-occurrence than with ASD. As ASD and ADHD might display overlapping symptoms, such as hyperfocus in ADHD, they can be somewhat challenging to separate. Research suggests that ADHD and ASD are related conditions with phenomenological and etiological overlap (Antshel & Russo, 2019). As such, attention towards adolescents with ADHD and GI is also important, as ADHD and ASD may show some overlap in symptoms. Further, in both ADHD and ASD individuals may show a rigidity or hyperfocus when it comes to GI that clinically needs to be explored to ensure the best support for the individual.

Furthermore, of the two assigned genders at birth, more AFAB had received one or more psychiatric diagnoses previously. The high number of participants who had one or more psychiatric diagnoses is consistent with other studies of adolescents that included a high percentage of AFAB with GI (Kaltiala-Heino et al., 2018). For example, one study from Finland found that 75% of applicants to the gender identity clinic were undergoing assessment for psychiatric diagnoses and that the group displayed psychiatric diagnoses similar to patients referred for psychiatric assessment without GI (Kaltiala-Heino et al., 2015). Depression and anxiety are the most common psychiatric diagnoses (Hilton et al., 2022; Holt et al., 2016; Kaltiala-Heino et al., 2018; Kaltiala et al., 2020; Kozlowska et al., 2021), which is consistent with this study. Another consistency with the present study is that self-harm and suicide attempts in gender-incongruent individuals are high, especially in AFAB, and typically in AFAB also having an ASD diagnosis (de Graaf et al., 2022; Hartig et al., 2022; Skagerberg et al., 2013). Autistic females with average IQs receive ASD diagnoses later than males. As a consequence, they might develop secondary mental health difficulties, and they are typically diagnosed with other psychiatric diagnoses before they receive an ASD assessment/diagnosis (Fusar-Poli et al., 2022; Lai & Baron-Cohen, 2015; Posserud et al., 2021).

Studies have pointed out that SRS results must be interpreted cautiously when differentiating between autism characteristics and social phobia (Cholemkery et al., 2014). Another study that uses the SRS and discusses mental health indicates that, based on self-report measures, gender-diverse children and adolescents are a particularly vulnerable group at risk of mental health needs (Mahfouda et al., 2019). The high number of psychiatric diagnoses in this nationally representative sample study based on standardized assessment confirms that adolescents experiencing GI have a higher risk of mental health needs. The minority stress model points out that increased stigmatization, prejudice, and discrimination create a hostile environment that can cause mental health issues. It also points out that lesbians, bisexuals, and gay men (LGBs) more frequently experience mental health concerns than heterosexuals (Meyer, 2003). The model has been applied to the gender-diverse population and stresses that gender-non-conforming youth are particularly vulnerable (Diamond, 2013).

### Clinical and Research Implications

Based on an overrepresentation of ASD diagnoses and autism characteristics in the GI population, we suggest that ASD screening should be systematically conducted in gender assessment clinics. Using standardized screening instruments such as the SRS seems valuable to the standard assessment of GI youths. The SRS is also easy to use and effective in capturing individuals’ social challenges in their everyday lives. By systematic screening with the SRS, autistic characteristics can be addressed early in the assessment phase, and clinicians can adjust their communication from the beginning (Strang et al., 2018). An ASD diagnosis may improve access to support and a better understanding of the individual strengths and challenges, and lead to a better quality of life. The high percentage of other psychiatric diagnoses and conditions indicates the need for a general psychiatric assessment and a biopsychosocial approach. Systematic screening for ASD in the AFAB group might be particularly important, as we know they often show other psychiatric symptoms before assessment for ASD is given. The findings in the current study should encourage clinicians to provide care that is informed by patients’ communication preferences and autism-related needs. The findings should not be interpreted to support additional gatekeeping or delays around access to assessment and gender-affirming treatment. Further controlled population-based studies using diagnostic criteria for ADHD and ASD are needed.

### Strengths and Limitations

All adolescents referred to the NGT being considered for GT have follow-up appointments at the service. The psychiatric assessments for GI adolescents in Norway are standardized with validated diagnostic tools, including both parent report of developmental history and interview with the adolescents. This is considered a strength of the current study. This study has some limitations. The sample size was small but similar to that of other GI clinical studies. The year of inclusion was the first year of the COVID 19 pandemic. There were generally lower-than-average attendance, more use of video consultations, and more participants who needed follow-up appointments than expected. This study is limited by the lack of information we have on the non-participants. Conclusions made from the American norm data need to be interpreted cautiously in a Norwegian sample. In the current study autistic characteristics are investigated using questionnaires completed by parents, not by self-report. Future studies should explore in greater depth GI adolescents’ own experience of social challenges and mental health needs, and involve non-binary youth.

### Ethics

Written consent was obtained from each individual, and the study was approved by the Data Inspectorate and the Regional Ethics Committee (*Declaration of Helsinki*, 2008). The study was carried out in accordance with the Declaration of Helsinki.

## References

[CR1] Achenbach, T. M. (2014). J. T. e. o. c. p. Achenbach system of empirically based assessment (ASEBA). 1–8. 10.1186/s13034-019-0291-2 PMC6610912 31312253

[CR2] American Psychiatric Association (2022). *Diagnostic and statistical manual of mental disorders* (5th ed., text rev.). 10.1176/appi.books.9780890425787.

[CR3] Antshel, K. M., & Russo, N. (2019). Autism Spectrum disorders and ADHD: Overlapping phenomenology, diagnostic issues, and treatment considerations. *Curr Psychiatry Rep*, *21*(5), 34. 10.1007/s11920-019-1020-5. 30903299 10.1007/s11920-019-1020-5

[CR4] Arnoldussen, M., de Rooy, F. B. B., de Vries, A. L. C., van der Miesen, A. I. R., Popma, A., & Steensma, T. D. (2022). Demographics and gender-related measures in younger and older adolescents presenting to a gender service. *European Child and Adolescent Psychiatry*. 10.1007/s00787-022-02082-8. 36370316 10.1007/s00787-022-02082-8 PMC10682114

[CR5] Atladottir, H. O., Gyllenberg, D., Langridge, A., Sandin, S., Hansen, S. N., Leonard, H., Gissler, M., Reichenberg, A., Schendel, D. E., & Bourke, J. (2015). The increasing prevalence of reported diagnoses of childhood psychiatric disorders: A descriptive multinational comparison. *European Child and Adolescent Psychiatry*, *24*(2), 173–183. 24796725 10.1007/s00787-014-0553-8

[CR6] Auyeung, B., Baron-Cohen, S., Ashwin, E., Knickmeyer, R., Taylor, K., & Hackett, G. (2009). Fetal testosterone and autistic traits. *British Journal of Psychology*, *100*(Pt 1), 1–22. 10.1348/000712608x311731. 18547459 10.1348/000712608X311731

[CR8] Baron-Cohen, S., Wheelwright, S., Skinner, R., Martin, J., & Clubley, E. (2001). The autism-spectrum quotient (AQ): Evidence from Asperger syndrome/high-functioning autism, males and females, scientists and mathematicians. *Journal of Autism and Developmental Disorders*, *31*(1), 5–17. 10.1023/a:1005653411471. 11439754 10.1023/a:1005653411471

[CR7] Baron-Cohen, S., Auyeung, B., Nørgaard-Pedersen, B., Hougaard, D. M., Abdallah, M. W., Melgaard, L., Cohen, A. S., Chakrabarti, B., Ruta, L., & Lombardo, M. V. (2015). Elevated fetal steroidogenic activity in autism. *Molecular Psychiatry*, *20*(3), 369–376. 10.1038/mp.2014.48. 24888361 10.1038/mp.2014.48 PMC4184868

[CR9] Bejerot, S., & Eriksson, J. M. (2014). Sexuality and gender role in autism spectrum disorder: A case control study. *PloS One*, *9*(1), e87961. 10.1371/journal.pone.0087961. 24498228 10.1371/journal.pone.0087961 PMC3909328

[CR10] Bewley, S., Clifford, D., McCartney, M., & Byng, R. (2019). *Gender incongruence in children, adolescents, and adults*. In: British Journal of General Practice. 10.3399/bjgp19X701909 PMC6428456 30923140

[CR14] Bölte, S., Neufeld, J., Marschik, P. B., Williams, Z. J., Gallagher, L., & Lai, M. C. (2023). Sex and gender in neurodevelopmental conditions. *Nature Reviews: Neurology*, *19*(3), 136–159. 10.1038/s41582-023-00774-6. 36747038 10.1038/s41582-023-00774-6 PMC10154737

[CR11] Bouman, W. P., de Vries, A. L., & T’Sjoen, G. (2016). *Gender dysphoria and gender incongruence: An evolving inter-disciplinary field*. In: Taylor & Francis. 10.3109/09540261.2016.1125740 26769232

[CR12] Bouzy, J., Brunelle, J., Cohen, D., & Condat, A. (2023). Transidentities and autism spectrum disorder: A systematic review. *Psychiatry Research*, *323*, 115176. 10.1016/j.psychres.2023.115176. 36996732 10.1016/j.psychres.2023.115176

[CR13] Brunissen, L., Rapoport, E., Chawarska, K., & Adesman, A. (2021). Sex differences in gender-diverse expressions and identities among youth with autism spectrum disorder. *Autism Research*, *14*(1), 143–155. 10.1002/aur.2441. 33283980 10.1002/aur.2441

[CR15] Cholemkery, H., Mojica, L., Rohrmann, S., Gensthaler, A., & Freitag, C. M. (2014). Can autism spectrum disorders and social anxiety disorders be differentiated by the social responsiveness scale in children and adolescents? *Journal of Autism and Developmental Disorders*, *44*(5), 1168–1182. 10.1007/s10803-013-1979-4. 24682652 10.1007/s10803-013-1979-4

[CR16] Constantino, J. N. G., C.P (2005). *The social responsiveness scale*. Western Psychological Services.

[CR17] de Graaf, N. M., Carmichael, P., Steensma, T. D., & Zucker, K. J. (2018). (2000–2017). *Journal of Sexual Medicine*, *15*(10), 1381–1383. 10.1016/j.jsxm.2018.08.002. Evidence for a change in the sex ratio of children referred for gender dysphoria: Data from the gender identity development service in London. 10.1016/j.jsxm.2018.08.002 30195563

[CR18] de Graaf, N. M., Steensma, T. D., Carmichael, P., VanderLaan, D. P., Aitken, M., Cohen-Kettenis, P. T., de Vries, A. L. C., Kreukels, B. P. C., Wasserman, L., Wood, H., & Zucker, K. J. (2022). Suicidality in clinic-referred transgender adolescents. *European Child and Adolescent Psychiatry*, *31*(1), 67–83. 10.1007/s00787-020-01663-9. 33165650 10.1007/s00787-020-01663-9

[CR19] de Vries, A. L. C., Noens, I. L. J., Cohen-Kettenis, P. T., van Berckelaer-Onnes, I. A., & Doreleijers, T. A. (2010). Autism spectrum disorders in gender dysphoric children and adolescents. *Journal of Autism and Developmental Disorders*, *40*(8), 930–936. 10.1007/s10803-010-0935-9. 20094764 10.1007/s10803-010-0935-9 PMC2904453

[CR20] Demetriou, E. A., Lampit, A., Quintana, D. S., Naismith, S. L., Song, Y. J. C., Pye, J. E., Hickie, I., & Guastella, A. J. (2018). Autism spectrum disorders: A meta-analysis of executive function. *Molecular Psychiatry*, *23*(5), 1198–1204. 10.1038/mp.2017.75. 28439105 10.1038/mp.2017.75 PMC5984099

[CR21] Dhejne, C. (2017). *On gender dysphoria* [Doctoral dissertation: Karolina Institutet].

[CR22] Diamond, L. M. (2013). Chapter 11—Sexual-minority, gender-nonconforming, and transgender youths. In D. S. Bromberg & W. T. O’Donohue (Eds.), *Handbook of Child and Adolescent Sexuality* (pp. 275–300). Academic Press. 10.1016/B978-0-12-387759-8.00011-8.

[CR23] Expósito-Campos, P., Gómez-Balaguer, M., Hurtado-Murillo, F., & Morillas-Ariño, C. (2023). Evolution and trends in referrals to a specialist gender identity unit in Spain over 10 years (2012–2021). *Journal of Sexual Medicine*, *20*(3), 377–387. 10.1093/jsxmed/qdac034. 36763946 10.1093/jsxmed/qdac034

[CR24] Fusar-Poli, L., Brondino, N., Politi, P., & Aguglia, E. (2022). Missed diagnoses and misdiagnoses of adults with autism spectrum disorder. *European Archives of Psychiatry and Clinical Neuroscience*, *272*(2), 187–198. 10.1007/s00406-020-01189-w. 32892291 10.1007/s00406-020-01189-w PMC8866369

[CR25] Hartig, A., Voss, C., Herrmann, L., Fahrenkrug, S., Bindt, C., & Becker-Hebly, I. (2022). Suicidal and nonsuicidal self-harming thoughts and behaviors in clinically referred children and adolescents with gender dysphoria. *Clinical Child Psychology and Psychiatry*, *27*(3), 716–729. 10.1177/13591045211073941. 35213240 10.1177/13591045211073941 PMC9234769

[CR26] Heylens, G., Aspeslagh, L., Dierickx, J., Baetens, K., Van Hoorde, B., De Cuypere, G., & Elaut, E. (2018). The co-occurrence of gender dysphoria and autism spectrum disorder in adults: An analysis of cross-sectional and clinical chart data. *Journal of Autism and Developmental Disorders*, *48*(6), 2217–2223. 10.1007/s10803-018-3480-6. 29427119 10.1007/s10803-018-3480-6

[CR27] Hilton, M. N., Boulton, K. A., Kozlowska, K., McClure, G., & Guastella, A. J. (2022). The co-occurrence of neurodevelopmental disorders in gender dysphoria: Characteristics within a paediatric treatment-seeking cohort and factors that predict distress pertaining to gender. *Journal of Psychiatric Research*, *149*, 281–286. 10.1016/j.jpsychires.2022.02.018. 35306277 10.1016/j.jpsychires.2022.02.018

[CR28] Hines, M. (2020). Human gender development. *Neuroscience and Biobehavioral Reviews*, *118*, 89–96. 10.1016/j.neubiorev.2020.07.018. 32707345 10.1016/j.neubiorev.2020.07.018 PMC10691233

[CR29] Holt, V., Skagerberg, E., & Dunsford, M. (2016). Young people with features of gender dysphoria: Demographics and associated difficulties. *Clinical Child Psychology and Psychiatry*, *21*(1), 108–118. 10.1177/1359104514558431. 25431051 10.1177/1359104514558431

[CR605] Huang, A. X., Hughes, T. L., Sutton, L. R., Lawrence, M., Chen, X., Ji, Z., & Zeleke, W. (2017). Understanding the Self in Individuals with Autism Spectrum Disorders (ASD): A Review of Literature. *Frontiers in Psychology, 8*, 1422–1422. 10.3389/fpsyg.2017.01422. 10.3389/fpsyg.2017.01422 PMC5572253 28878717

[CR65] 10.1037/t15169-0003.

[CR30] Hull, L., Lai, M. C., Baron-Cohen, S., Allison, C., Smith, P., Petrides, K. V., & Mandy, W. (2019). Gender differences in self-reported camouflaging in autistic and non-autistic adults. *Autism*, *24*(2), 352–363. 10.1177/1362361319864804. 31319684 10.1177/1362361319864804

[CR31] Idring, S., Lundberg, M., Sturm, H., Dalman, C., Gumpert, C., Rai, D., Lee, B. K., & Magnusson, C. (2015). Changes in prevalence of autism spectrum disorders in 2001–2011: Findings from the Stockholm youth cohort. *Journal of Autism and Developmental Disorders*, *45*(6), 1766–1773. 25475364 10.1007/s10803-014-2336-y

[CR32] Isaksen, J., Diseth, T. H., Schjølberg, S., & Skjeldal, O. H. (2012). Observed prevalence of autism spectrum disorders in two Norwegian counties. *European Journal of Paediatric Neurology*, *16*(6), 592–598. 10.1016/j.ejpn.2012.01.014. 22342070 10.1016/j.ejpn.2012.01.014

[CR33] Kallitsounaki, A., & Williams, D. M. (2022). Autism spectrum disorder and gender dysphoria/incongruence. A systematic literature review and meta-analysis. *Journal of Autism and Developmental Disorders*. 10.1007/s10803-022-05517-y. 35596023 10.1007/s10803-022-05517-y PMC10313553

[CR36] Kaltiala, R., Bergman, H., Carmichael, P., de Graaf, N. M., Egebjerg Rischel, K., Frisén, L., Schorkopf, M., Suomalainen, L., & Waehre, A. (2020). Time trends in referrals to child and adolescent gender identity services: A study in four nordic countries and in the UK. *Nordic Journal of Psychiatry*, *74*(1), 40–44. 10.1080/08039488.2019.1667429. 31556776 10.1080/08039488.2019.1667429

[CR35] Kaltiala-Heino, R., Sumia, M., Tyolajarvi, M., & Lindberg, N. (2015). Two years of gender identity service for minors: Overrepresentation of natal girls with severe problems in adolescent development. *Child Adolesc Psychiatry Ment Health*, *9*, 9. 10.1186/s13034-015-0042-y. 25873995 10.1186/s13034-015-0042-y PMC4396787

[CR34] Kaltiala-Heino, R., Bergman, H., Tyolajarvi, M., & Frisen, L. (2018). Gender dysphoria in adolescence: Current perspectives. *Adolesc Health Med Ther*, *9*, 31–41. 10.2147/ahmt.s135432. 29535563 10.2147/AHMT.S135432 PMC5841333

[CR37] Kaufman, J., Birmaher, B., Brent, D., Rao, U., Flynn, C., Moreci, P., Williamson, D., & Ryan, N. J. (1997). J. o. t. A. A. o. C., & Psychiatry, A. Schedule for affective disorders and schizophrenia for school-age children-present and lifetime version (K-SADS-PL): Initial reliability and validity data. *36*(7), 980–988. 10.1097/00004583-199707000-00021 9204677

[CR38] Kozlowska, K., McClure, G., Chudleigh, C., Maguire, A. M., Gessler, D., Scher, S., & Ambler, G. R. (2021). Australian children and adolescents with gender dysphoria: Clinical presentations and challenges experienced by a multidisciplinary team and gender service. *Human Systems*, 26344041211010777. 10.1177/26344041211010777.

[CR39] Kreukels, B. P., & Guillamon, A. J. I. R. o. P. (2016). Neuroimaging studies in people with gender incongruence. *28*(1), 120–128. https://www.tandfonline.com/doi/pdf/10.3109/09540261.2015.1113163?needAccess=true. 10.3109/09540261.2015.1113163 26766406

[CR40] Lai, M. C., & Baron-Cohen, S. (2015). Identifying the lost generation of adults with autism spectrum conditions. *Lancet Psychiatry*, *2*(11), 1013–1027. 10.1016/s2215-0366(15)00277-1. 26544750 10.1016/S2215-0366(15)00277-1

[CR41] Lai, M. C., Lombardo, M. V., & Baron-Cohen, S. (2014). *Autism Lancet*, 383(9920), 896–910. 10.1016/s0140-6736(13)61539-1. 24074734 10.1016/S0140-6736(13)61539-1

[CR42] Loomes, R., Hull, L., & Mandy, W. P. L. (2017). What is the male-to-female ratio in autism spectrum disorder? A systematic review and meta-analysis. *Journal of the American Academy of Child and Adolescent Psychiatry*, *56*(6), 466–474. 10.1016/j.jaac.2017.03.013. 10.1016/j.jaac.2017.03.013 28545751

[CR43] Lord, C., Rutter, M., DiLavore, P. C., Risi, S., Gotham, K. O., & Bishop, S. L. (2012). *Autism Diagnostic Observation schedule, Second Edition (ADOS-2) Manual (Part I): Modules 1–4*. Western Psychological Services.

[CR44] Lundström, S., Mårland, C., Kuja-Halkola, R., Anckarsäter, H., Lichtenstein, P., Gillberg, C., & Nilsson, T. (2019). Assessing autism in females: The importance of a sex-specific comparison. *Psychiatry Research*, *282*, 112566. 10.1016/j.psychres.2019.112566. 31558402 10.1016/j.psychres.2019.112566

[CR48] Maenner, M. J., Warren, Z., Williams, A. R., Amoakohene, E., Bakian, A. V., Bilder, D. A., Durkin, M. S., Fitzgerald, R. T., Furnier, S. M., Hughes, M. M., Ladd-Acosta, C. M., McArthur, D., Pas, E. T., Salinas, A., Vehorn, A., Williams, S., Esler, A., Grzybowski, A., Hall-Lande, J., & Shaw, K. A. (2023). Prevalence and characteristics of Autism Spectrum Disorder among children aged 8 years - Autism and Developmental Disabilities Monitoring Network, 11 sites, United States, 2020. *MMWR: Surveillance Summaries*, *72*(2), 1–14. 10.15585/mmwr.ss7202a1. 10.15585/mmwr.ss7202a1 PMC10042614 36952288

[CR49] Mahfouda, S., Panos, C., Whitehouse, A. J. O., Thomas, C. S., Maybery, M., Strauss, P., Zepf, F. D., O’Donovan, A., van Hall, H. W., Saunders, L. A., Moore, J. K., & Lin, A. (2019). Mental health correlates of autism spectrum disorder in gender diverse young people: Evidence from a specialised child and adolescent gender clinic in Australia. *Journal of Clinical Medicine*, *8*(10), 1503. 10.3390/jcm8101503. 31547002 10.3390/jcm8101503 PMC6832530

[CR50] Mazzoli, F., Cassioli, E., Ristori, J., Castellini, G., Rossi, E., Cocchetti, C., Romani, A., Angotti, T., Giovanardi, G., Mosconi, M., Lingiardi, V., Speranza, A. M., Ricca, V., Vignozzi, L., Maggi, M., & Fisher, A. D. (2022). Apparent autistic traits in transgender people: A prospective study of the impact of gender-affirming hormonal treatment. *Journal of Endocrinological Investigation*, *45*(11), 2059–2068. 10.1007/s40618-022-01835-1. 35779204 10.1007/s40618-022-01835-1 PMC9525411

[CR51] Meyer, I. H. (2003). Prejudice, social stress, and mental health in lesbian, gay, and bisexual populations: Conceptual issues and research evidence. *Psychological Bulletin*, *129*(5), 674–697. 10.1037/0033-2909.129.5.674. 12956539 10.1037/0033-2909.129.5.674 PMC2072932

[CR52] Nobili, A., Glazebrook, C., Bouman, W. P., Baron-Cohen, S., & Arcelus, J. (2020). The stability of autistic traits in transgender adults following cross-sex hormone treatment. *International Journal of Transgender Health*, *21*(4), 431–439. 10.1080/26895269.2020.1783738. 34993521 10.1080/26895269.2020.1783738 PMC8726673

[CR71] Øien, R. A., Cicchetti, D. V., & Nordahl-Hansen, A. (2018). Gender dysphoria, sexuality and autism spectrum disorders: A systematic map review. *Journal of Autism and Developmental Disorders*, *48*(12), 4028–4037. 30019279 10.1007/s10803-018-3686-7

[CR53] Posserud, M. B., Solberg, S., Engeland, B., Haavik, A., J., & Klungsøyr, K. (2021). Male to female ratios in autism spectrum disorders by age, intellectual disability and attention-deficit/hyperactivity disorder. *Acta Psychiatrica Scandinavica*, *144*(6), 635–646. 10.1111/acps.13368. 34494265 10.1111/acps.13368

[CR55] Russell, I., Pearson, B., & Masic, U. (2021). A longitudinal study of features associated with autism spectrum in clinic referred, gender diverse adolescents accessing puberty suppression treatment. *Journal of Autism and Developmental Disorders*, *51*(6), 2068–2076. 10.1007/s10803-020-04698-8. 32936414 10.1007/s10803-020-04698-8

[CR54] Rutter, M., Le Couteur, A., & Lord, C. (2003). *Autism Diagnostic interview-revised (ADI-R)*. Western Psychological Services.

[CR56] Sachser, C., Berliner, L., Holt, T., Jensen, T. K., Jungbluth, N., Risch, E., Rosner, R., & Goldbeck, L. (2017). International development and psychometric properties of the child and adolescent trauma screen (CATS). *J J o a d*, *210*, 189–195. 10.1016/j.jad.2016.12.040 28049104

[CR58] Skagerberg, E., Parkinson, R., & Carmichael, P. (2013). Self-harming thoughts and behaviors in a group of children and adolescents with gender dysphoria. *International Journal of Transgenderism*, *14*(2), 86–92.

[CR57] Skagerberg, E., Di Ceglie, D., & Carmichael, P. (2015). Brief report: Autistic features in children and adolescents with gender dysphoria. *Journal of Autism and Developmental Disorders*, *45*(8), 2628–2632. 10.1007/s10803-015-2413-x. 25772537 10.1007/s10803-015-2413-x

[CR59] Strang, J. F., Meagher, H., Kenworthy, L., de Vries, A. L. C., Menvielle, E., Leibowitz, S., Janssen, A., Cohen-Kettenis, P., Shumer, D. E., Edwards-Leeper, L., Pleak, R. R., Spack, N., Karasic, D. H., Schreier, H., Balleur, A., Tishelman, A., Ehrensaft, D., Rodnan, L., Kuschner, E. S., Mandel, F., & Anthony, L. G. (2018). Initial clinical guidelines for co-occurring autism spectrum disorder and gender dysphoria or incongruence in adolescents. *Journal of Clinical Child and Adolescent Psychology*, *47*(1), 105–115. 10.1080/15374416.2016.1228462. 27775428 10.1080/15374416.2016.1228462

[CR60] Surén, P., Havdahl, A., Øyen, A. S., Schjølberg, S., Reichborn-Kjennerud, T., Magnus, P., Bakken, I. J. L., & Stoltenberg, C. (2019). Diagnosing autism spectrum disorder among children in Norway. *Tidsskrift for den Norske Laegeforening*, *139*(14), 10.4045/tidsskr.18.0960(Diagnostisering av autismespekterforstyrrelser hos barn i Norge.). 10.4045/tidsskr.18.0960 31592612

[CR600] Thrower, E., Bretherton, I., Pang, K. C., Zajac, J. D., & Cheung, A. S. (2020). Prevalence of Autism Spectrum Disorder and Attention-Deficit Hyperactivity Disorder Amongst Individuals with Gender Dysphoria: A Systematic Review. *Journal of Autism and Developmental Disorders, 50*(3), 695–706. 10.1007/s10803-019-04298-1 10.1007/s10803-019-04298-1 31732891

[CR61] van der Miesen, A. I. R., de Vries, A. L. C., Steensma, T. D., & Hartman, C. A. (2018a). Autistic symptoms in children and adolescents with gender dysphoria. *Journal of Autism and Developmental Disorders*, *48*(5), 1537–1548. 10.1007/s10803-017-3417-5. 29189919 10.1007/s10803-017-3417-5 PMC5889781

[CR62] van der Miesen, A. I. R., Hurley, H., Bal, A. M., & de Vries, A. L. C. (2018b). Prevalence of the wish to be of the opposite gender in adolescents and adults with autism spectrum disorder. *Archives of Sexual Behavior*, *47*(8), 2307–2317. 10.1007/s10508-018-1218-3. 29736809 10.1007/s10508-018-1218-3 PMC6245048

[CR164] Warrier, V., Greenberg, D. M., Weir, E., Buckingham, C., Smith, P., Lai, M. C., Allison, C., & Baron-Cohen, S. (2020). Elevated rates of autism, other neurodevelopmental and psychiatric diagnoses, and autistic traits in transgender and gender-diverse individuals. *Nat Commun, 11*(1), 3959. 10.1038/s41467-020-17794-1 10.1038/s41467-020-17794-1 PMC7415151 32770077

[CR64] Wechsler, D. (2008). *Wechsler Adult Intelligence Scale–Fourth Edition*. (WAIS-IV).APA PsycTests.

[CR63] Wechsler, D. J. B., & Pearson, M. N. (2014). *Wechsler intelligence scale for children–Fifth Edition*. WISC-V).

[CR66] World Health Organization (1993). *ICD-10 for Mortality and Morbidity Statistics*.

[CR67] World Health Organization (2018). *ICD-11 for Mortality and Morbidity Statistics (2018) Release version*

[CR68] World Health Organization. https://icd.who.int/browse11/l-m/en#/http://id.who.int/icd/entity/90875286.

[CR69] World Medical Association. *Declaration of Helsinki. Ethical Principles for Medical Research Involving Human Subjects* (2008). https://www.wma.net/policies-post/wma-declaration-of-helsinki-ethical-principles-for-medical-research-involving-human-subjects/. 10.1191/0969733002ne486xx 16010903

[CR70] Zucker, K. J. (2017). Epidemiology of gender dysphoria and transgender identity. *Sexual Health*, *14*(5), 404–411. 10.1071/sh17067. 28838353 10.1071/SH17067

